# Improving fiscal space for health from the perspective of efficiency in low- and middle-income countries: What is the evidence?

**DOI:** 10.7189/jogh.10.020421

**Published:** 2020-12

**Authors:** Wu Zeng, Yao Yao, Hélène Barroy, Jonathan Cylus, Guohong Li

**Affiliations:** 1 School of Nursing & Health Studies, Georgetown University, Washington D.C., USA; 2 Shanghai Jiao Tong University School of Medicine, Shanghai, China; 3 China Hospital Development Institute, Shanghai Jiao Tong University, Shanghai, China; 4 World Health Organization, Geneva, Switzerland; 5 European Observatory on Health Systems and Policies, London, UK; 6 London School of Economics and Political Science, London, UK; 7 London School of Hygiene & Tropical Medicine, London, UK

## Abstract

**Background:**

Conceptual frameworks of fiscal space for health have traditionally considered health system efficiency improvements as a means to free up resources for the sector. However, there has been no comprehensive review of the evidence to confirm the relationship between efficiency and fiscal space.

**Methods:**

We conducted a systematic review to synthesize evidence on whether efficiency gains increase fiscal space for health. We searched bibliographic databases for specific keywords – namely, fiscal space, efficiency and health – and identified 22 articles that examined links between efficiency gains and fiscal space for health. The articles, which encapsulated 28 case studies, were included in the analysis.

**Results:**

The 28 case studies varied widely with regard to how efficiency was evaluated, the extent to which efficiency was explored, and how efficiency gains could be achieved. Half of the studies assessed both technical and allocative efficiency, and the other half assessed technical efficiency only. The indicators to examine potential inefficiencies varied substantially among studies. The most frequently cited inefficiencies stemmed from public financial management (budget implementation, budget allocation and strategic purchasing) and governance issues, even though these were characterized in various ways. The second most cited set of inefficiencies that caused health systems to function poorly were those related to health service delivery. Procurement and delivery of input factors was also mentioned in some studies as a source of inefficiency. Though most studies conceded that efficiency gains were a potential means to improve fiscal space for health, very few quantified the potential gains or explored practical mechanisms to translate efficiency gains into fiscal space for health.

**Conclusions:**

While the conceptual link between efficiency gains and fiscal space for health may be assumed, there is no direct empirical evidence proving that efficiency gains translate into more resources for the health sector. Mechanisms to translate efficiency gains into fiscal space are barely explored in the fiscal space literature. Public financial management rules and related rules for reallocating funds within the sector need to be further examined to guide countries in the transformation of efficiency gains into more resources for health.

There is a growing recognition of the importance of creating fiscal space for health in many low- and middle-income countries (LMICs) in order to achieve universal health coverage (UHC) and health-related Sustainable Development Goals (SDGs). In the 2010 World Health Report, strengthening health system financing was highlighted as a means to achieve UHC [1,2]. Increasing fiscal space for health can have a significant impact on the ability of governments to achieve adequate and sustainable health financing, particularly in health systems which are primarily financed by government budgets [3,4].

Fiscal space refers to budgetary flexibility that allows a government to provide additional resources for a particular public purpose without impacting fiscal sustainability [5]. Previous studies have suggested that fiscal space may be expanded through a number of mechanisms, including: (1) conducive macroeconomic conditions; (2) budget reprioritization towards health; (3) new earmarked taxation sources; (4) efficiency gains; and (5) external resources [6,7].

The fiscal space for health framework was developed in 2010 [6]. Since then, many LMICs have assessed fiscal space for health and reviewed fiscal space in relation to the five mechanisms of potential expansion above [8]. Some of the findings from these assessments indicate that significant space can be generated through efficiency gains [9] – if the same level of outputs could be achieved with a smaller level of inputs, more resources could be made available and reallocated within the sector. However, research on efficiencies in the health sector has not been effectively folded into the existing literature on fiscal space for health. For example, in the fiscal space for health literature there is little guidance given on how to assess causes of inefficiencies or how to quantify the potential monetary benefits of addressing inefficiencies. More importantly, few literature defining efficiency and inefficiency in the health sector or the measures that may lead to efficiencies or inefficiencies was effectively leveraged in the literature on fiscal space for health [10,11]. There has also been little exploration of how and under what circumstances efficiency gains could be converted to government budgets for health, requiring clearer guidance in understanding potential drivers of government budgets for health, for which the concept of budgetary space for health has been developed [12].

A more systematic examination of efficiency in the fiscal space for health literature is necessary, especially given the increasingly prominent role that efficiency gains as a tool for generating fiscal space now plays in policy dialogues and given the diversity of approaches used in fiscal space analyses examining the relationship between efficiency gains and fiscal space [8]. This study aims to systematically synthesize the evidence available on efficiency gains to increase fiscal space for health, in an effort to identify the inefficiencies and associated policy responses that could enhance fiscal space and to guide future work related to the links between efficiency and fiscal space.

## METHODS

### Analytical approach

**Figure 1:**
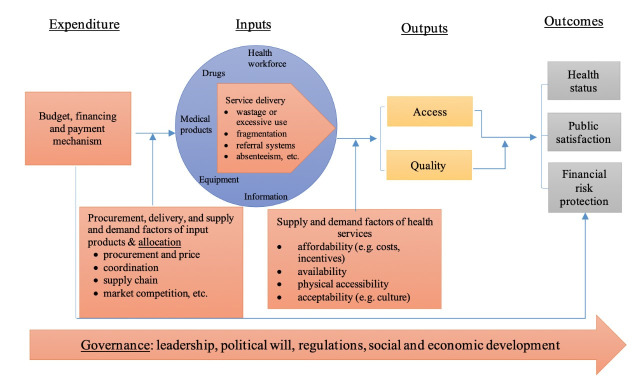
Theoretical framework to analyse causes of inefficiencies in health systems.

This review concentrates on the relationship between efficiency and fiscal space for health. It builds on existing frameworks to identify causes of inefficiencies in health systems and uses a systematic approach to classify potential causes of inefficiencies and efficiencies that have already been identified in the fiscal space for health literature. In the last few years, several frameworks to identify efficiencies and inefficiencies have emerged, including the health system framework [1] and input-output-outcome framework [13]. Additionally, some tools have been developed to collect data for examining health system inefficiencies [14]. **Figure 1** shows the health system and input-output-outcome frameworks in one consolidated framework. This framework is similar to the one used by Yip and Hafez [15], but provides more detail on where efficiencies and inefficiencies could arise and where they must be more closely examined. Our intention was not to generate a new framework for analysing health system inefficiencies but rather to create a framework with which we could classify the potential causes of efficiencies and inefficiencies that existed within the fiscal space for health literature.

Our review initially followed the input-output-outcome framework, in which factors beyond inputs and outputs –what are called external factors or environmental determinants of the performance – are suggested to be examined to identify efficiencies and inefficiencies. We thus added two sets of external factors as potential causes of efficiencies and inefficiencies: (1) procurement and delivery of input products as well as supply and demand factors influencing input factors, and (2) supply and demand factors on access to care using the care utilization framework [16]. These two sets of external factors, added to the three factors from the input-output-outcome framework – governance, service delivery, and budgeting and financing – constituted a complete set of potential efficiencies and inefficiencies.

The red boxes in **Figure 1** represent the factors that create efficiencies and inefficiencies through their impact on how funding is allocated and used, and the conversion of inputs into health care outputs. These efficiencies and inefficiencies can be categorized under five domains: (1) governance (eg, corruption, transparency, and accountability); (2) factors associated with budget allocation and execution processes, also known as public financial management (PFM); (3) procurement, delivery, supply and demand factors of inputs; (4) supply and demand factors of health services; and (5) factors related to the use of inputs once procured (eg, mix of health workers, medical products, equipment, use of referral systems, and use of information for monitoring and decision making). The five domains are interrelated, which create both efficiencies and inefficiencies.

### Desk search strategy

To identify articles for review, we searched four major electronic bibliographic databases on public health and economics for combinations of three terms: (1) fiscal space; (2) efficiency; and (3) health. The initial search was conducted on 22 September 2018 on PubMed, EconLit, Academic Search Premier, and Web of Science, and updated on 25 October 2018. We also conducted a search for grey literature through the POPLINE database and Google Scholar. All searches were conducted in English. The initial search identified 6248 non-duplicate publications that were eligible for title and abstract screening, including 14 articles shared by the World Health Organization (WHO). The research team reviewed all records from the Google Scholar search, checked their eligibility and compared them to those obtained from electronic databases. No additional articles were included from the Google Scholar search.

### Exclusion criteria

The search records were uploaded in Endnote X8 and independently screened by two reviewers. After eliminating duplicate records, the titles and abstracts of the remaining studies were reviewed to assess their relevance based on four criteria: (1) studies examining fiscal space for health in a country or a region; (2) studies examining efficiency as a means to increase fiscal space; (3) studies assessing fiscal space using the World Bank framework; and (4) studies conducted in LMICs. Articles that met or possibly met the criteria were included in the full-text review. Articles were excluded if they met any of the following exclusion criteria: (1) studies not related to health, including social protection; (2) studies that provided no analysis of fiscal space; (3) studies that did not address efficiency of health systems; (4) studies synthesizing previous fiscal space analyses; and (5) studies published prior to 2000. The full text review, which included 53 articles, was conducted independently by Wu Zeng and Yao Yao. Thirty-three articles were excluded based on the exclusion criteria. **Table 1** lists the reasons for exclusion. References from the remaining 20 articles were reviewed, and two more articles were added from the reference review to the final list of articles selected. The 22 articles included 28 case studies.

**Table 1:** Reasons for article exclusion

Number of articles	Reason for exclusion
11	Commentary
2	Synthesizing previous studies
3	Duplication
14	Not related to health
2	Note related to efficiency of health systems
1	Not conducted in LMIC

LMIC – low- and middle-income country

### Data collection and extraction

The data collected from each article included the basic characteristics of each study (ie, publication year, topic, type of article, assessment approach including the type of efficiency and efficiency assessment method), as well as which components of efficiency and fiscal space the articles addressed (ie, efficiency indicators, key inefficiency identified, proposed approaches to address the inefficiency, feasibility and scope of fiscal space from efficiency gains, and estimates of fiscal space from efficiency). The data was collected in an Excel spreadsheet. For each article, the above-mentioned information was extracted. Data extraction was carried out primarily by one researcher. A second researcher reviewed and checked the collected data.

### Data synthesis

The analysis of the data collected in the Excel spreadsheet (Microsoft Inc, Seattle, WA, USA) focused on two dimensions. The first dimension was related to the characteristics of the overall study, including the efficiency assessment methods and the type of efficiency. Researchers often distinguish between two types of efficiency, technical and allocative. Technical efficiency refers to achieving maximum output (ie, number of health services) for a given level of inputs (ie, human resources, equipment and drugs), while allocative efficiency refers to choosing the appropriate mix of inputs to achieve desired outputs [6,15,17].

The second dimension was related to specific findings on efficiency and fiscal space, including the efficiency indicators used in the assessment, the key inefficiency identified, the proposed approaches to address inefficiencies, the feasibility and scope for fiscal space, and the estimated fiscal space for health gains from efficiencies. Efficiency indicators were grouped into three categories: (1) general efficiency indicators that capture a country’s health outcomes, such as life expectancy, infant mortality rate and overall mortality rate; (2) allocative efficiency indicators that provide information on the allocation of resources; and (3) technical efficiency indicators that concern the outputs produced for given inputs, such as bed occupancy rate, average length of stay and efficiency scores from data envelopment analysis (DEA) or stochastic frontier analysis (SFA). At times, it was hard to draw a clear line between the efficiency indicator categories. Key inefficiencies were grouped into four categories, based on the approach described in **Figure 1** with PFM mixed with governance: (1) PFM, strategic purchasing and governance; (2) procurement and delivery of input factors; (3) service delivery (use and organization of input factors); and (4) demand and supply factors of health services.

## RESULTS

**Figure 2:**
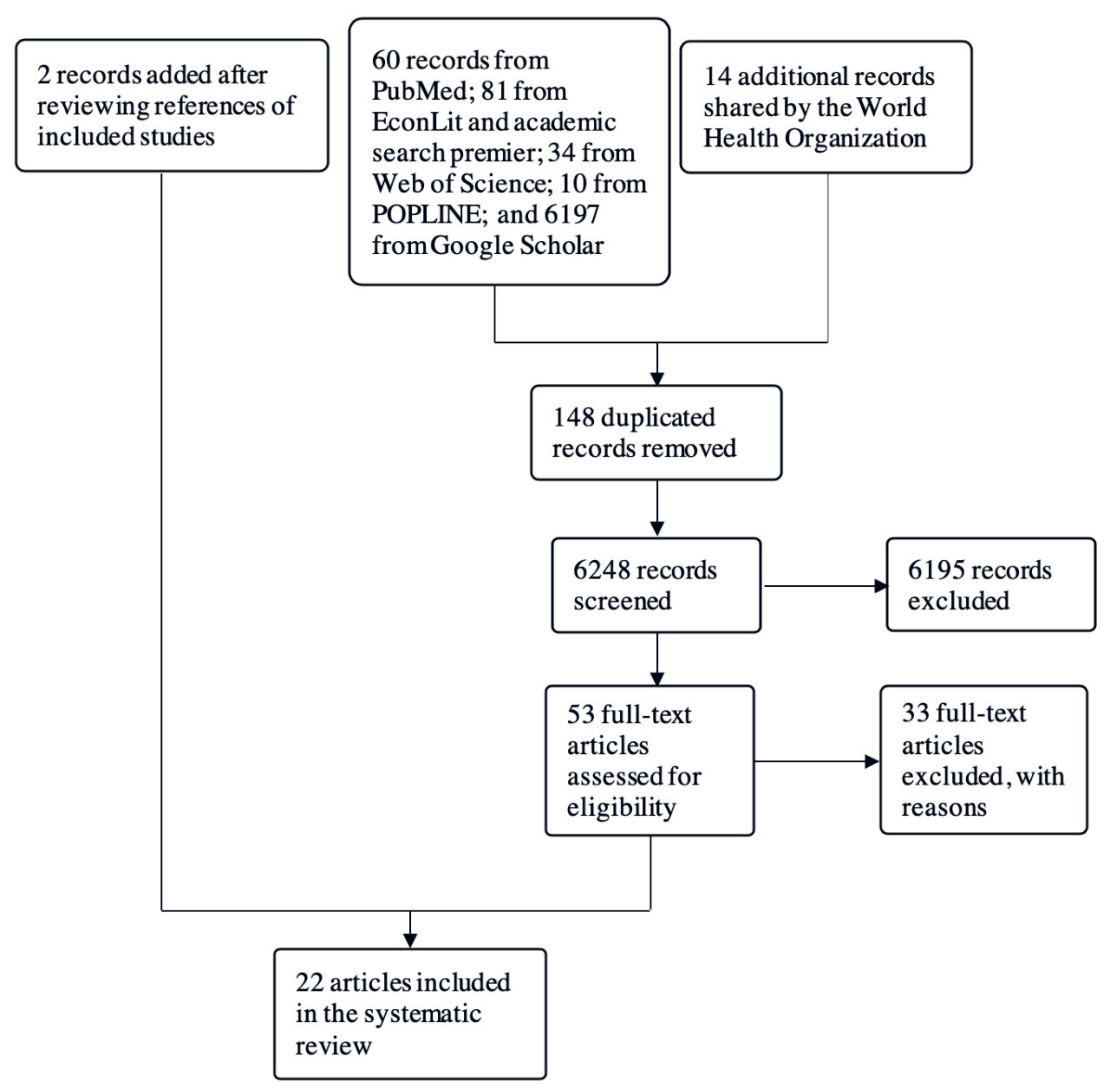
Flow diagram for study identification.

Twenty-two articles with 28 case studies were deemed eligible for this review, based on our database search, our desktop research, and consultations with the WHO. The flow diagram in **Figure 2** shows how eligible articles were identified.

### Overview of studies

Of the 28 selected case studies from the 22 articles identified, five were obtained from peer review articles, and the rest were grey reports. Twenty-seven of the case studies were published after 2010; one had unknown publication dates. Twenty-five of the case studies were conducted for specific countries: two studies were conducted in Ghana, Indonesia, and Uganda, respectively, and one study was conducted in each of the following countries, Afghanistan, Bangladesh, Bhutan, Cambodia, Cote d’Ivoire, Guinea, India, Kenya, Liberia, Nepal, Nigeria, Peru, Rwanda, South Africa, Sudan, United Republic of Tanzania, Tonga, Ukraine, and Zimbabwe. The three remaining studies were regional, two focused on a group of sub-Saharan Africa countries and one on a group of countries in the Americas. Geographically, there were 16 studies conducted in Africa, two in the Americas, eight in Asia, one in Oceania, and one in Eastern Europe. Half of the 28 case studies (14) assessed both technical and allocative efficiency, the other half assessed technical efficiency (TE) only, and none focused solely on allocative efficiency (AE). There were four studies that used both quantitative and qualitative approaches to assess efficiencies, 19 studies used a qualitative assessment only, and five studies used only a quantitative approach. **Table 2** provides details of the selected studies.

**Table 2:** General characteristics of selected studies

Institutional author or last name of first author	Publication year	Country	Region	Language	Topic	Type of article	Type of efficiency	Efficiency assessment method
Belay [18]	2015	Nepal	Asia	English	General health	Report	TE and AE	Both quantitative and qualitative analysis (DEA)
Ichoku [19]	2015	Nigeria	Africa	English	General health	Journal article	TE only	Qualitative
James [20]	2014	Tanzania	Africa	English	General health	Report	TE only	Both quantitative and qualitative (DEA)
Kioko [21]	2013	Kenya	Africa	English	General health	Report	TE only	Quantitative (DEA)
Levin [22]	Unknown	Cote d'Ivoire	Africa	English	HIV	Report	TE only	Qualitative
Mathonnat [23]	2010	Sub-Saharan Africa	Africa	English	General health	Report	TE only	Quantitative (DEA)
Ministry of Public Health [24]	2017	Afghanistan	Asia	English	General health	Report	TE and AE	Qualitative
Mohammed [25]	2016	Sudan	Africa	English	Health insurance fund	Journal article	TE only	Both quantitative and qualitative analysis (regression)
Novignon [26]	2015	Sub-Saharan African	Africa	English	General health	Report	TE only	Quantitative (DEA and SFA)
Novignon [27]	2017	Ghana	Africa	English	Primary care	Journal article	TE only	Quantitative (SFA)
Okwero [28]	2010	Uganda	Africa	English	General health	Report	TE only	Quantitative (regression)
Pan American Health Organization (PAHO) [29]	2015	Americas	Americas	English	General health	Report	TE only	Qualitative
PAHO [30]	2015	Peru	Americas	English	General health	Report	TE and AE	Qualitative
Regondi [31]	2012	South Africa	Africa	English	General health	Journal article	TE only	Qualitative
Schieber [32]	2012	Ghana	Africa	English	General health	Report	TE only	Qualitative
Sharma [33]	2016	Bhutan	Asia	English	General health	Journal article	TE only	Both quantitative and qualitative (regression)
Tandon [6]	2010	Cambodia	Asia	English	General health	Report	TE and AE	Qualitative
Tandon [6]	2010	India	Asia	English	General health	Report	TE and AE	Qualitative
Tandon [6]	2010	Indonesia	Asia	English	General health	Report	TE and AE	Qualitative
Tandon [6]	2010	Rwanda	Africa	English	General health	Report	TE and AE	Qualitative
Tandon [6]	2010	Tonga	Oceania	English	General health	Report	TE and AE	Qualitative
Tandon [6]	2010	Uganda	Africa	English	General health	Report	TE and AE	Qualitative
Tandon [6]	2010	Ukraine	Eastern Europe	English	General health	Report	TE and AE	Qualitative
World Bank [34]	2017	Zimbabwe	Africa	English	General health	Report	TE and AE	Qualitative
World Bank [35]	2016	Liberia	Africa	English	General health	Report	TE and AE	Qualitative
World Bank [36]	2016	Bangladesh	Asia	English	General health	Report	TE only	Qualitative
World Bank [37]	2009	Indonesia	Asia	English	General health	Report	TE and AE	Qualitative
World Bank [38]	2015	Guinea	Africa	French	General health	Report	TE and AE	Qualitative

### Efficiency indicators

The selected studies used a variety of indicators to quantify inefficiencies in health systems and health service delivery. High-level efficiency indicators tended to be health outcome indicators, such as life expectancy, healthy life years, infant mortality rate (IMR), and maternal mortality rate (MMR). Some studies examined variations in these indicators across countries, regions, or districts, which were used as evidence of existing inefficiencies. There were also studies that investigated variations in health outputs, not outcomes, across different geographic areas, which served as an indicator of technical inefficiencies in health systems. The most common output indicators were: immunization coverage, skilled birth attendance, and unmet need for contraceptives. At the hospital level, common efficiency indicators were average length of stay, bed occupancy rate, and bed turnover rate. Other technical efficiency indicators included absenteeism rate, staff vacancy rate, health personnel density, and availability of drugs and equipment, each of which also quantifies the availability and adequacy of input factors. A few studies used either DEA or SFA, and at times both, to estimate health system and health service delivery efficiency scores; these scores were then used as a technical efficiency indicator. One study used costs per hospital stay and costs per hospital day as an efficiency indicator. Allocative efficiency indicators mostly measured the allocation of budget by type of service, level of care, function of input factors (eg, personnel wages, administration), and disease burden.

### Major inefficiencies and interventions to address inefficiencies

Table S1 in the **Online Supplementary Document** provides detailed information on key inefficiencies identified in the fiscal space for health literature and categorizes proposed interventions from the 28 cases studies. Of the 28 case studies, 22 cited PFM/governance issues as a source of inefficiency, five studies did not explore causes of inefficiencies, and one study explored inefficiency but did not mention PFM/governance as a cause.

The second most cited source of health system malfunctions was inefficiency in health service delivery. Of the 28 case studies, 16 (57.1%) addressed inefficiencies in health service delivery. These inefficiencies were often related to the availability and use of inputs, including human resources, medical products, pharmaceuticals, equipment, and health information, as well as the way inputs were organized and how the referral system functioned. The most common inefficiency concerns related to service delivery were: absenteeism (Indonesia, Nepal, Tanzania), ghost workers (Tanzania, Uganda), understaffing (Cote d’Ivoire, Nepal, Tanzania, Zimbabwe), and shortage of drugs and equipment (Bangladesh, Bhutan). Other inefficiency concerns included excessive use of resources, such as extended hospital stays in Ukraine, and an oversupply of staff, for example in Liberia and Afghanistan.

Only four of the 28 studies mentioned issues with the procurement and delivery of inputs as a source of inefficiency (Afghanistan, Bhutan, Tanzania, Zimbabwe). Low procurement capacity was one of the reasons cited as a cause of poor health system performance. In Bhutan, the drug market was identified as a source of inefficiency, with small pharmaceutical markets and a heavy reliance on imported medicines leading to high drug prices and creating vulnerability in the drug supply chain. The studies included little exploration of inefficiencies related to the supply and demand of health services, such as accessibility, affordability, acceptability of health services, or quality of care.

Some studies proposed interventions to address efficiency concerns. According to our assessment, nine (32.1%) of the 28 studies described potential interventions to tackle health system inefficiencies in detail. Half of the studies (14), provided directional or generic recommendations, which may not be useful guidance for policy-makers seeking to improve the performance of their health systems or to create fiscal space for health. Five studies (17.8%) did not provide any recommendation.

The proposed interventions could be grouped under the same four categories as the inefficiencies themselves: 1) PFM, strategic purchasing and governance; (2) procurement and delivery of input factors; (3) service delivery (use and organization of input factors); and (4) demand and supply factors of health services. The key proposed PFM and governance interventions included performance-based payments, provider payment reforms, and streamlined budget preparation, allocation and execution. The Nepal study, for example, highlighted provider payment reforms as a means to strengthen health system performance.

### Feasibility, scope and estimates of fiscal space from efficiency gains

The studies were generally optimistic about the possibility of creating fiscal space through efficiency gains. Of the 28 case studies, half (14) indicated that countries could free up sizable resources for health by addressing inefficiencies. The Guinea study, for example, described significant potential for the country to create fiscal space through efficiency gains. Two (7.1%) studies (Afghanistan, Rwanda) pointed to moderate potential for efficiency gains to generate fiscal space for budgeting, while three (10.7%) studies (Bangladesh, Liberia, Peru) indicated limited scope for efficiency gains in those countries. The nine (32.1%) remaining studies did not assess the scope of efficiency gains for creating fiscal space (see Table****S1 in the **Online Supplementary Document**).

Only seven studies (25%) quantified the financial gains that could be generated by addressing inefficiencies. The remaining 21 studies (75%) provided no estimate of potential gains that could be potentially used for health. In the seven studies which attempted to quantify gains, most failed to provide complete estimates of the potential gains of addressing key inefficiencies. Some estimates were based on addressing absenteeism or length of stay only. Others focused on service delivery inefficiencies only. The potential savings estimates were expressed in monetary units, as a share of gross domestic product, or as a share of government budget or expenditure (see Table S1 in the **Online Supplementary Document**).

No study explored how potential gains could be translated into more resources for the sector or how countries could overcome barriers within PFM systems that prevent countries from reallocating savings generated by efficiency gains. Other evidence suggests that efficiency often translated into budget cuts [39].

## DISCUSSION

This study synthesizes evidence on whether efficiency gains produce more fiscal space for health. Twenty-eight case studies across five regions were analysed as part of the study. Of the case studies that assess the potential scope of fiscal space gained by reducing inefficiencies, about half conclude that efficiency gains could significantly improve government fiscal space for health. Efficiency improvements should therefore be considered alongside domestic resource generation as a means to improve the health financing situation in a country.

The indicators used to evaluate efficiency varied widely among countries. In the fiscal space for health assessments, three broad types of efficiency indicators are used to measure fiscal space. The first focuses on variations in key health outcomes (eg, life expectancy, infant mortality rate), which are a result of both allocative and technical efficiencies. The second focuses on resource allocation organized by different characteristics (eg, population groups, levels of care). The third focuses on technical efficiency, such as the production of outputs for a given set of resources or the level or number of inputs for a given level of outputs (eg, length of stay, bed occupancy rate, unit costs of health services). Not all of the studies which assess the scope of fiscal space gained through efficiency improvements examine all three types of efficiencies. Many examine technical efficiency only. Some concentrate on the technical efficiency of health service delivery only, as opposed to the health system as a whole. The indicators for each type of efficiency also vary across studies. For example, in the South Africa study, the general indicators used are IMR and MMR [31]. Whereas in the Peru study, the general indicators are life expectancy and healthy years of life [30]. The indicators for measuring allocative and technical efficiencies vary even more substantially across the studies. Developing a standard and essential set of efficiency indicators would help to standardize measurement.

The most appropriate methods with which to measure health care and health system efficiency are unaddressed in the assessments, as well as how efficiency metrics could be used to inform policy and managerial decisions. Most studies (19/28 case studies) use qualitative descriptions of health statistics to demonstrate inefficiencies in the health system or to synthesize prior studies. Only one study conducted focus group discussions or stakeholder informant interviews to systematically and qualitatively assess efficiency concerns [20]. Most quantitative studies used DEA, SFA or regression analysis to examine efficiencies in the health care or health delivery system. Such analyses generally provide an understanding of overall efficiency but often fail to identify the causes of inefficiency. In failing to explain the causes of inefficiency, some studies which use DEA or SFA significantly compromise the validity of the analysis and thereby limit the use of the findings to develop meaningful and practical interventions to address efficiency concerns.

Only a handful of inefficiencies are explored in the existing fiscal space for health literature. Those that are examined tend to be related to PFM and health service delivery. The WHO estimated that 20%-40% of health resources are wasted [1]. The same WHO report also identified the top ten causes of health system wastage, including the use of counterfeit drugs, limited use of generic drugs, overuse of equipment, unnecessary investigations and procedures, an inappropriate skills mix among personnel, medical errors and suboptimal quality of care. The WHO findings are consistent with some of the inefficiencies found in fiscal space assessments. Inconsistencies in the types of inefficiencies identified are to be expected but the concentration of inefficiencies related to PFM and health service delivery suggests that many fiscal space inefficiencies are being missed in the literature. Very few studies explore inefficiencies related to the procurement of medicines or equipment, or on supply and demand factors that affect procurement and the ability of patients to receive care (eg, physical accessibility of health facilities). Very few studies looked at administrative inefficiencies, such as waste or leakages caused by fraud or from the misuse of funds. Allocative efficiency – across sectors and within sectors – is also rarely addressed in the fiscal space for health studies, although it is a fundamental concept in discussions on efficiency [40]. One reason why inefficiencies may have been overlooked in the literature is because of a lack of data.

Not all studies in this review included recommendations to address specific inefficiencies. Those that did often did not always match recommendations to the causes of health system inefficiencies. The failure to provide recommendations may be a result of the initial scope of work agreed upon as part of the fiscal space analysis. In the future, all fiscal space analyses should include space for recommendations, which could then be used to help design targeted interventions and evidence-based policies. Although many studies state that efficiency gains could generate substantial scope for fiscal space, only seven of the 28 studies in this review estimated budgetary space in monetary terms (eg, potential savings from reduced length of stay in hospital). None of the studies systematically and comprehensively quantified potential budgetary space. Modelling gains in fiscal space requires specific sets of data on each identified inefficiency. For inefficiencies that are common across countries and prevalent in LMICs, a uniform model to quantify efficiency gains in monetary terms would help to standardize fiscal space estimates. Though the scope of work of fiscal space analyses will vary substantially from country to country, it would be useful for all analyses to include data collection that would help quantify potential budgetary space. This would, in turn, provide policy-makers with more detailed and accurate information.

The most important omission in the studies reviewed is the lack of direct evidence on how fiscal space would be expanded by addressing inefficiencies, making more funds available for reinvestment in the health sector. No study provided information on whether financial gains actually translated into fiscal space for the health sector. Instead, the studies remained theoretical, assessing potential but not actual gains and failing to explore how funds transform into fiscal space or expanded financial room (ie, where and how to incorporate the money that is saved by addressing inefficiencies). While the link between fiscal space and efficiency is conceptually valid, there is increasing empirical evidence showing that the link is not automatic. The fiscal space analysis from efficiency gains seems to assume that (1) improved efficiency results in more money for the health sector; (2) money generated through technical efficiency gains will stay in the health sector; and (3) identifying sources of inefficiency will automatically lead to more fiscal space. However, in practice, efficiency improvements do not necessarily mean spending less and, therefore, don’t guarantee that more resources will be made available for the health sector. This is the result of a number of factors: (1) addressing inefficiencies may require additional investments [41]; (2) some evidence shows that better efficiency can actually translate into budget cuts for the sector [8,9]; and (3) the transformation process is far from automatic and requires at least four steps – identifying inefficiencies, implementing corrective actions, reallocating sector resources, and quantifying the resources made available by addressing inefficiencies. Improving efficiency can enhance fiscal space, but there are cases when it is not used to that end. Although efficiency improvements have the potential to expand fiscal space in many countries, there is no direct evidence on the degree to which fiscal space could be expanded by improving health system efficiencies in health systems.

We must acknowledge that health system efficiency does not necessarily expand fiscal space for health. This is critical for LMICs to understand as they endeavour to create fiscal space and move towards UHC. Health system efficiency should be examined more thoroughly and systematically, especially how to ensure that efficiency gains lead to an increase in fiscal space instead of leading to budget cuts for the health sector. Despite the lack of direct evidence, efficiency improvements remain an important element of every country’s effort to enhance their health system. In some countries, addressing efficiency concerns could be a starting point for raising financial resources within the health sector or advocating for a greater share of the overall budget. Improving health system efficiency could not only save resources, which could be reinvested in the health sector, but it could also strengthen the relationship between finance and health authorities and help trigger broader health system reform.

## Additional material

Online Supplementary Document
