# Supplementary material for: Improving fiscal space for health from the perspective of efficiency in low- and middle-income countries: What is the evidence?
Source: J Glob Health. 2020 Sep 21;10(2):020421. doi: 10.7189/jogh.10.020421 (PMC7568933; doi:10.7189/jogh.10.020421)
Supplement: Online Supplementary Document [file jogh-10-020421-s001.pdf]

Table S1. Characteristics of cost-effectiveness analysis of included studies

Institutional author or last name of first author	Country	Efficiency indicators	Key inefficiencies	Proposed approaches to address inefficiencies	Feasibility and scope for fiscal space	Potential fiscal space from efficiency gains
Belay[18]	Nepal	**1. General indicator**<br>1.1. Life expectancy<br>**2. Technical efficiency indicator**<br>2.1. Efficiency score from DEA, with input of district income, and output of life expectancy<br>2.2. Bed turnover rate<br>2.3. Bed occupancy rate<br>2.4. Average length of stay<br>2.5. Coverage of skilled birth attendance<br>2.6. Coverage of diphtheria-tetanus-pertussis (DTP3) immunization	**1. PFM, strategic purchasing and governance**<br>1.1. Inefficiency of spending and accountability<br>**2. Service delivery**<br>2.1. Absenteeism<br>2.2. Understaffing<br>2.3. Stockouts of medicines	**1. PFM, strategic purchasing and governance**<br>1.1. Link payment to performance to promote transparency and accountability<br>1.2. Change provider payment to per diem system<br>1.3. Provide conditional grants to hospitals and districts with global budget<br>1.4. Consolidate incentive programmes to enhance monitoring<br>1.5. Subsidize consumers rather than suppliers<br>**2. Service delivery**<br>2.1. Address absenteeism<br>**3. Procurement and delivery of input factors**<br>3.1. Improve drug procurement:<br>3.1.1. Procure drug centrally<br>3.1.2. Split tenders<br>3.2. Improve storage and distribution of drugs<br>3.3. Improve maintenance of equipment	Good	Addressing absenteeism would save 1.6% of the government budget. Splitting tenders would save 18% of the procurement value.

Institutional author or last name of first author	Country	Efficiency indicators	Key inefficiencies	Proposed approaches to address inefficiencies	Feasibility and scope for fiscal space	Potential fiscal space from efficiency gains
Ichoku[19]	Nigeria	**1. Allocative efficiency indicator**<br>1.1. Share of capital budget between Ministry of Health (MoH) and implementation agencies<br>**2. Technical efficiency indicator**<br>2.1. Efficiency score using DEA and SFA	1.**PFM, strategic purchasing and governance**<br>1.1. Lack of symmetry between responsibility and resource allocation; large amount of resources was controlled by the MoH<br>1.2. Waste and unnecessary expenditure within headquarters<br>1.3. Corruption and a lack of transparency and accountability<br>2.**Service delivery**<br>2.1. Inefficient service delivery	**1. PFM, strategic purchasing and governance**<br>1.1. Improve governance, and anti-corruption<br>1.2. Improve absorption capacity by adding quality health workforce	No assessment	No estimation
James[20]	Tanzania	**1. Technical efficiency indicator**<br>1.1. Efficiency scores	**1.PFM, strategic purchasing and governance**<br>1.1. Disburse funds to health facilities that are not operational or do not exist<br>1.2. Most disbursements to hospitals towards the last quarter of the financial year<br>**2.Service delivery**<br>2.1. Staff shortage and maldistribution<br>2.2. Staff productivity and efficiency<br>2.3. Skills shortage and lack of training<br>2.4. Existence of ghost workers<br>2.5. The referral system does not function appropriately<br>**3.Procurement, delivery of input factors**<br>3.1. Disbursement of drug budget is unpredictable<br>3.2. Development partners procure medical commodities for the MoH, but are uncoordinated and inefficient<br>3.3. Some critical medications not centrally procured	**1. PFM, strategic purchasing and governance**<br>1.1. Stop allocating funds to facilities that do not exist and are not operational<br>1.2. Avoid disbursing large amounts of funding near the last quarter of the financial year<br>1.3. Improve budget forecasting<br>1.4. Improve accountability and eliminate complicated disbursement procedures<br>1.5. Improve information and transparency<br>**2. Service delivery**<br>2.1. Eliminate ghost workers<br>2.2. Improve human resource management and supervision<br>2.3. Build capacity<br>2.4. Improve the functionality of referral system	Good	Efficiency improvements could lead to TSh1.1 billion in savings per year by 2024-2025

Institutional author or last name of first author	Country	Efficiency indicators	Key inefficiencies	Proposed approaches to address inefficiencies	Feasibility and scope for fiscal space	Potential fiscal space from efficiency gains
			3.4. Delay and maldistribution of drugs to health facilities	2.5. Integrate vertical programmes<br>**3. Procurement and delivery of medicines**<br>3.1. Coordinate the procurement of medical commodities<br>3.2. Eliminate inefficiencies in medical supply delivery		
Kioko[21]	Kenya	**1. Technical efficiency indicator**<br>1.1. Efficiency scores, with inputs including clinical officers, nurses and non-medical personnel and beds, and outputs including outpatient visits and inpatient admissions	No exploration. The average efficiency was 72.6% for hospitals (constant returns to scale), 50.7% for health centres, and 43% for dispensaries.	No specific recommendation	No assessment	No estimation
Levin[22]	Cote d'Ivoire	**1. Allocative efficiency indicator**<br>1.1. Share of expenditure on management and administration<br>**2. Technical efficiency indicator**<br>2.1. Health personnel occupancy rate	**1.PFM, strategic purchasing and governance**<br>1.1. High management and administrative costs<br>**2.Service delivery**<br>2.1. Shortage of health personnel<br>2.2. Insufficient health clinics	**1. General recommendations**<br>1.1. Evaluate inefficiencies in the health sector, particularly in administration and management	Good	No estimation
Mathonnat[23]	Sub-Saharan Africa	**1. Technical efficiency indicator**<br>1.1. Efficiency scores	No exploration. The average efficiency score ranged from 56%-59%.	**1. PFM, strategic purchasing and governance**<br>1.1. Improve budget execution rates, increase efficiency of public and private spending<br>1.2. Strengthen coordination	Good	No estimation
Ministry of Public Health[24]	Afghanistan	**1. Allocative efficiency indicator**<br>1.1. Burden of disease and budget allocation<br>1.2. Share of spending on preventive care<br>**2. Technical efficiency indicator**	1.**PFM, strategic purchasing and governance**<br>1.1. Corruption<br>1.2. Low budget execution<br>1.3. Spending on preventive care is low<br>1.4. Lack of power in budget and staffing	1. **PFM, strategic purchasing and governance**<br>1.1. Strengthen transparency and accountability<br>1.2. Strengthen strategic purchasing of health services	Moderate	Efficiency improvements could save US$36.2 million from 2017-2021, which is

Institutional author or last name of first author	Country	Efficiency indicators	Key inefficiencies	Proposed approaches to address inefficiencies	Feasibility and scope for fiscal space	Potential fiscal space from efficiency gains
		2.1. Budget execution rate<br>2.2. Bed occupancy rate<br>2.3. Average length of stay<br>2.4. Ratio of health personnel to hospital beds	at hospitals<br>**2.Service delivery**<br>2.1. Low efficiency at hospitals<br>2.2. Relatively higher number of medical doctors in hospitals<br>2.3. Lack of capacity of procurement and hospital management	1.3. Increase autonomy for hospitals and improve operational capacity<br>2. **Service delivery**<br>2.1. Enhance task-shifting<br>2.2. Provide training on procurement<br>2.3. Develop public-private partnerships		equivalent to US$7.2 million per year.
Mohammed[25]	Sudan	**1. General indicator**<br>1.1. Infant mortality compared to other countries<br>1.2. Governance indicator<br>**2. Technical efficiency indicator**<br>2.1. Drug expenditure as share of health expenditure	**1.Service delivery**<br>1.1. Inefficiency in health-care service distribution<br>**2.PFM, strategic purchasing and governance**<br>2.1. Absent of regulating policies	No specific recommendation	No assessment	No estimation
Novignon[26]	Sub-Saharan Africa	**1. Technical efficiency indicator**<br>1.1. Efficiency scores, based on DEA and SFA, with inputs including per capita health expenditure and GDP per capita, and outputs including DPT3, IMR, and percentage of population using an improved sanitation facility	No exploration. The average efficiency scores ranged from 45%-72% using different models.	No specific recommendation	Good	Sub-Saharan African countries could save up to 0.10% per capita health expenditure and 0.08% of GDP per capita.
Novignon[27]	Ghana	**1. Technical efficiency indicator**<br>1.1. Efficiency scores, with outpatient visits as an output and the number of personnel, hospital beds, expenditure on other capital items and administration as inputs.	No exploration. The average efficiency score was 51% for health centres.	**1. PFM, strategic purchasing and governance**<br>1.1. Link finance to performance measures	No assessment	Efficiency improvements could save facilities about GH₵ 11,450.70 (US$7633.80).

Institutional author or last name of first author	Country	Efficiency indicators	Key inefficiencies	Proposed approaches to address inefficiencies	Feasibility and scope for fiscal space	Potential fiscal space from efficiency gains
Okwero[28]	Uganda	**1. General efficiency indicator**<br>1.1. Variation of life expectancy<br>**2. Technical efficiency indicator**<br>2.1. Variation of output indicators, including DPT3, and institutional delivery across districts	No exploration	**1. PFM, strategic purchasing and governance**<br>1.1. Clarify the concept of on-budget project support and commit to its consistent application during the budgeting process<br>1.2. Establish a system to capture and monitor large ticket inputs from off-budget donor support<br>1.3. Pursue available avenues for improved donor coordination and harmonization<br>**2. Procurement and delivery of input factors (medicine and human resources)**<br>2.1. Harmonize procurement of third-party commodities<br>2.2. Clarify and agree on the roles of main stakeholders involved in drug procurement and logistics management<br>2.3. Review government acts on procurement to reduce procurement restrictions<br>2.4. Review drug financing through primary health centres and credit lines to avoid duplication<br>2.5. Remove obstacles that impede basic personnel management functions<br>2.6. Institute measures to attract and retain staff, especially in	Good	No estimation

Institutional author or last name of first author	Country	Efficiency indicators	Key inefficiencies	Proposed approaches to address inefficiencies	Feasibility and scope for fiscal space	Potential fiscal space from efficiency gains
				rural and remote areas.<br>2.7. Align sector performance to output and results		
PAHO[29]	Americas	No specific indicator	**1. PFM, strategic purchasing, and governance**<br>1.1. Segmentation and fragmentation of health insurance system<br>1.2. Poor coordination mechanisms between different organizations and care levels<br>1.3. No regulatory framework<br>1.4. Lack of incentives to providers	**1. PFM, strategic purchasing and governance**<br>1.1. Reduce segmentation and fragmentation of insurance providers<br>1.2. Strengthen regulation and incentives for providers<br>1.3. Introduce case-mix measurement systems	No assessment	No estimation
PAHO[30]	Peru	**1. General indicator**<br>1.1. Life expectancy<br>1.2. Healthy life years	**1. PFM, strategic purchasing and governance**<br>1.1. Difficulties in executing regional budgets	No specific recommendation	Limited	No estimation
Regondi[31]	South Africa	**1. General indicator**<br>1.1. IMR and MMR<br>**2. Technical efficiency indicator**<br>2.1. Length of stay<br>2.2. Bed utilization rate<br>2.3. Tuberculosis (TB) cure rates	**1. PFM, strategic purchasing and governance**<br>1.1. Weak decentralization to the district level<br>1.2. Weak stewardship at the central level and a plethora of vertical health programmes overwhelming managers at the local level<br>1.3. Inappropriate distribution of resources between different levels of care<br>1.4. Low level of monitoring of spending	**1. PFM, strategic purchasing and governance**<br>1.1. Clarify responsibility at different levels of government to reduce duplication and ensure greater accountability<br>1.2. Reconsider the distribution of resources to the low level of service delivery points<br>1.3. Institute results-based financing (RBF)<br>**2. Service delivery**<br>2.1. Integrate HIV/AIDS services into broader health systems;	No assessment	No estimation

Institutional author or last name of first author	Country	Efficiency indicators	Key inefficiencies	Proposed approaches to address inefficiencies	Feasibility and scope for fiscal space	Potential fiscal space from efficiency gains
				2.2. Use step-down and community-based approaches for delivering HIV/AIDS care and treatment<br>2.3. Reduce anti-retroviral (ARV) tender prices using generic drugs		
Schieber[32]	Ghana	**1. Allocative efficiency indicator**<br>1.1. Share of National Health Insurance Scheme (NHIS) expenditure to fund MoH and Ghana Health Service<br>**2. Technical efficiency indicator**<br>2.1. Higher salaries among health staff<br>2.2. Share of individuals in the top two wealth quintiles exempted from paying NHIS	**1. PFM, strategic purchasing and payment reform**<br>1.1. Poorly planned investment in hospital infrastructure<br>1.2. Low productivity and high administrative costs of health facilities<br>1.3. Orientation towards curative rather than primary and preventive care<br>1.4. Eligibility for subsidy; some eligibility goes to wealthy groups<br>1.5. NHIS provider payment systems; diagnosis-related group (DRG) without cap<br>1.6. Operational inefficiencies; delays in claims processing and difficulty to monitor expenditure flows<br>**2. Service delivery**<br>2.1. Payment to medicines: major issues concern not just prices and spending but quality, prescribing patterns, fraud, abuse, and, most critically, patient	No specific recommendation	No assessment	No estimation

Institutional author or last name of first author	Country	Efficiency indicators	Key inefficiencies	Proposed approaches to address inefficiencies	Feasibility and scope for fiscal space	Potential fiscal space from efficiency gains
			health outcomes			
Sharma[33]	Bhutan	**1. General indicator**<br>1.1. Variation in life expectancy across countries<br>**2. Technical efficiency indicator**<br>2.1. Variation of costs of outpatient department<br>2.2. Variation of institutional delivery<br>2.3. Variation of unmet need for conception<br>2.4. Variation of length of stay at hospitals	**1. PFM, strategic purchasing and governance**<br>1.1. Unconditional grants to districts and hospitals<br>1.2. Rigid budget line reporting<br>**2. Procurement, delivery, supply and demand factors associated with input factors**<br>2.1. Small pharmaceutical market and dependence on imported medical supplies<br>2.2. Relatively high price of drugs and medical supplies<br>2.3. Vulnerability of drug supplies (shortage)	**1. PFM, strategic purchasing and governance**<br>1.1. Link financing to performance<br>1.2. Move away from budget line reporting<br>**2. Procurement of input factors**<br>2.1. Collaborate with regional countries and international agencies to ensure stability in drug procurement	No assessment	No estimation

Institutional author or last name of first author	Country	Efficiency indicators	Key inefficiencies	Proposed approaches to address inefficiencies	Feasibility and scope for fiscal space	Potential fiscal space from efficiency gains
Tandon[6]	Rwanda	No specific indicator	**1. PFM, strategic purchasing and governance**<br>1.1. Misalignment between how budget resources are allocated and the cost estimates for priority programmes<br>1.2. Mismatch between donor funding allocations and national priorities	**1. PFM, strategic purchasing and governance**<br>1.1. Improve alignment of available domestic resources and international, donor-funded resources with needs and priorities outlined in Rwanda's Health Sector Strategic Plan	Moderate	No estimation
Tandon[6]	Uganda	**1. Allocative efficiency indicator**<br>1.1. Share of earmarked funding in the health budget (85%)<br>**2. Technical efficiency indicator**<br>2.1. Absenteeism rate<br>2.2. Ghost worker rate	**1. PFM, strategic purchasing and governance**<br>1.1. Inflexibility in the use of budget funding and donor resources<br>1.2. Leakages in primary health care conditional grants<br>1.3. Financial mismanagement (unjustified expenditure)<br>**2. Service delivery**<br>2.1. Waste in pharmaceutical sector<br>2.2. Absenteeism (37%)<br>2.3. Ghost workers (1.5%)	**1. PFM, strategic purchasing and governance**<br>1.1. Address the rigidities in budget financing and external donor funding through financial management systems<br>1.2. Strengthen the link between health expenditure and health programme outputs<br>1.3. Improve transparent procurement processes, and monitoring and accountability measures	Good	An estimated 13% of government health spending
Tandon[6]	Cambodia	No specific indicator	**1. PFM, strategic purchasing and governance**<br>1.1. Lack of adequate systems for tracking the use of public resources for health<br>1.2. Limited budgetary information for health facilities; lack of accountability in the use of health facility budgets<br>1.3. A lack of facility-level budgeting often leads to in-kind payments to facilities with fuel and materials	**1. PFM, strategic purchasing and governance**<br>1.1. Modernize public finance systems<br>1.2. Improve planning and tracking of financial resource allocations<br>1.3. More closely align funding with the programmes in the health sector strategy	Good	No estimation

Institutional author or last name of first author	Country	Efficiency indicators	Key inefficiencies	Proposed approaches to address inefficiencies	Feasibility and scope for fiscal space	Potential fiscal space from efficiency gains
Tandon[6]	India	**1. General indicator**<br>1.1. Variation in health outcomes across states<br>**2. Allocative efficiency indicator**<br>2.1. 60% of National Rural Health Mission (NRHM) funds tied to 15 different fragmented programmes<br>2.2. 70% of state health budgets are consumed by salaries and non-discretionary spending<br>**3. Technical efficiency indicator**<br>3.1. Absenteeism in public sector primary health centres ranged from 40%-50%	**1. PFM, strategic purchasing and governance**<br>1.1. More than 70% of state health budgets were consumed by salaries, at the expense of medicines, equipment, and other direct inputs into patient care<br>1.2. Leakage and corruption<br>**2. Service delivery**<br>2.1. Absenteeism in public sector primary health centres ranged from 40%-50%	**1. PFM, strategic purchasing and governance**<br>1.1. Encourage NRHM to focus on a smaller number of interventions that can be scaled up and are important for the health and financial protection of the poor	Good	No estimation
Tandon[6]	Indonesia	**1. Technical efficiency indicator**<br>1.1. Unspent reserves account for 3.1% of GDP<br>1.2. Absenteeism rate at primary health facilities was 40%	**1. PFM, strategic purchasing and governance**<br>1.1. The majority of district level spending is non-discretionary<br>1.2. Funding flows are fragmented<br>1.3. Limited absorption capacity	**1. PFM, strategic purchasing and governance**<br>1.1. Streamline the flow of funds to the subnational level<br>1.2. Improve absorptive capacity at the local level<br>1.3. Use incentives in the context of purchasing under the mandatory health insurance system<br>1.4. Improve governance<br>**2. Service delivery**<br>2.1. Address issues such as absenteeism among public health workers	Good	No estimation

Institutional author or last name of first author	Country	Efficiency indicators	Key inefficiencies	Proposed approaches to address inefficiencies	Feasibility and scope for fiscal space	Potential fiscal space from efficiency gains
Tandon[6]	Ukraine	**1. General indicator**<br>1.1. Corruption perception index<br>**2. Technical efficiency indicator**<br>2.1. Length of hospital stay<br>2.2. Share of primary care providers	**1. PFM, strategic purchasing and governance**<br>1.1. Numerous PFM rigidities<br>1.2. Structure and financing of the system are normative-based rather than needs-based<br>1.3. Local governments operate within a stringent intergovernmental fiscal framework<br>1.4. High recurrent spending, particularly for wages<br>1.5. Corruption results in unproductive spending and informal payments<br>**2. Service delivery**<br>2.1. High average length of hospital stays<br>2.2. No clear distinction between primary and secondary care<br>2.3. Excessive number of specialists	**1. PFM, strategic purchasing and governance**<br>1.1. Address rigidities and inefficiencies, as well as corruptions<br>**2. Service delivery**<br>2.1. Reduce the excessive hospital capacity and reorient the system towards primary health care<br>2.2. Address high informal payments	Good	Generate 0.34% GDP per year in fiscal space by reducing length of stay
Tandon[6]	Tonga	**1. Allocative efficiency indicator**<br>1.1. Expenditure on public health and preventive services as share of total government expenditure (less than 5%)<br>1.2. Share of expenditure on pharmaceuticals for noncommunicable diseases (60.5%)	**1. PFM, strategic purchasing and governance**<br>1.1. Limited expenditure on public health and preventive care<br>1.2. Excessive expenditure on pharmaceuticals for noncommunicable diseases<br>**2. Service delivery**<br>2.1. Late diagnosis of noncommunicable diseases, which incur expensive treatment<br>2.2. Excessive use of hospitals	**1. PFM, strategic purchasing and governance**<br>1.1. Improve absorptive capacity for additional resources<br>1.2. Shift resources to primary care<br>1.3. Strengthen the management of chronic diseases	Good	No estimation

Institutional author or last name of first author	Country	Efficiency indicators	Key inefficiencies	Proposed approaches to address inefficiencies	Feasibility and scope for fiscal space	Potential fiscal space from efficiency gains
World Bank[34]	Zimbabwe	**1. Allocative efficiency indicator**<br>1.1. Disease burden<br>1.2. Allocation of government budget between curative and preventive care<br>**2. Technical efficiency indicator**<br>2.1. Unmatched budget allocation and budget expenditure<br>2.2. Health worker density<br>2.3. Health worker vacancy rate<br>2.3. Availability of essential medicines at health facilities	**1. PFM, strategic purchasing and governance**<br>1.1. Excessive spending on curative care<br>1.2. High concentration of resources at hospital level<br>1.3. Lack of transparency, accountability and sound management of public revenue, expenditure and liabilities<br>1.4. Large amount of budget released towards the end of the financial year<br>**2. Service delivery**<br>2.1. Shortage of key human resources for health<br>2.2. Low availability of key diagnostic items<br>2.3. Low hospital performance, with low bed occupancy rate<br>**3. Procurement and delivery of input factors**<br>3.1. Weak procurement systems<br>3.2. Stockout of essential medicines<br>3.3. High increase of minimal wage	**1. PFM, strategic purchasing and governance**<br>1.1. Improve budget processes, from planning to execution and implementation<br>1.2. Implement programme-based budgeting<br>1.3. Link finance to performance	No assessment	No estimation
World Bank[35]	Liberia	**1. Technical efficiency indicator**<br>1.1. Bed occupancy rate<br>1.2. Cost per inpatient day<br>1.3. Cost per inpatient stay<br>1.4. Average length of stay	**1. Service delivery**<br>1.1. Insufficient input factors (eg, diagnostic equipment)<br>1.2. Overstaffed in some facilities while understaffed in others<br>1.3. Hospital inefficiency in providing care	**1. Service delivery**<br>1.1. Hold hospitals accountable for actual costs and outcomes of admitted patients	Limited	No estimation

Institutional author or last name of first author	Country	Efficiency indicators	Key inefficiencies	Proposed approaches to address inefficiencies	Feasibility and scope for fiscal space	Potential fiscal space from efficiency gains
World Bank[36]	Bangladesh	**1. Allocative efficiency indicator**<br>1.1. Budget execution rate<br>1.2. Budget and expenditure comparison<br>**2. Technical efficiency indicator**<br>2.1. Absenteeism rate<br>2.2. Annual consultants per doctor<br>2.3. Annual doctor consultants per capita<br>2.4. Availability of drugs and equipment<br>2.5. Bed occupancy rate and turnover rate<br>2.6. Average length of stay	**1. PFM, strategic purchasing and governance**<br>1.1. Budget formulation is fragmented, with two separate units within the Ministry of Health and Family Welfare preparing two separate budgets<br>1.2. Budget allocation process is not well-linked to the health needs of the population<br>**2. Service delivery**<br>2.1. Low salary for entry-level medical officers<br>2.2. Absenteeism among providers<br>2.3. Inadequate equipment in public health facilities<br>2.4. Stockout of essential drugs<br>2.5. Gaps in providing basic and comprehensive emergency obstetric care	**1. PFM, strategic purchasing and governance**<br>1.1. Ensure robust mechanisms for budget formulation and execution<br>**2. Service delivery**<br>2.1. Strengthen accountability through government mechanisms and civil society engagement<br>2.2. Examine public budget flows to identify areas of mismanagement and fraud	Limited	No estimation
World Bank[37]	Indonesia	**1. Allocative efficiency indicator**<br>1.1. Public health expenditure by levels of government<br>**2. Technical efficiency indicator**<br>2.1. Coverage of DPT3 across countries<br>2.2. Coverage of skilled birth attendance	**1. PFM, strategic purchasing and governance**<br>1.1. Public health expenditure is dominated by spending on personnel salaries<br>1.2. District health spending remains, for the most part, nondiscretionary<br>**2. Service delivery**<br>2.1. 40% absenteeism rate among medical workers	**1. PFM, strategic purchasing and governance**<br>1.1. Design interfiscal transfer<br>1.2. Improve coordination across all levels of government<br>**2. Service delivery**<br>2.1. Reevaluate incentives and governance issues	No assessment	No estimation
World Bank[38]	Guinea	**1. General indicator**<br>1.1. Maternal mortality rate<br>**2. Allocative efficiency indicator**<br>2.1. Budget allocation for basic health-care programmes	**1.PFM, strategic purchasing and governance**<br>1.1. Limited resources are not always allocated according to need<br>1.2. No funding priority is given to core	**1. PFM, strategic purchasing and governance**<br>1.1. Increase budget execution rate<br>1.2. Improve allocation of resources	Very high	No estimation

Institutional author or last name of first author	Country	Efficiency indicators	Key inefficiencies	Proposed approaches to address inefficiencies	Feasibility and scope for fiscal space	Potential fiscal space from efficiency gains
		2.2. Budget for central bureaucracy	public health programmes with a high level of impact capacity and positive externalities<br>1.3. Excessive resources for central administration<br>**2. Service delivery**<br>2.1. Health personnel are usually concentrated in urban areas	1.3. Decentralize authorities to local level<br>1.4. Financing not only inputs but also performance<br>**2. Service delivery**<br>2.1. Revitalize and generalize the community approach<br>2.2. Price quality and regulation of medicine<br>2.3. Develop human resource policies		

Notes: MMR: maternal mortality rate; IMR: infant mortality rate; DPT3: diphtheria-tetanus-pertussis; NHIS: National Health Insurance Scheme; DRG: diagnosis-related group; ARV: anti-retroviral; TB: tuberculosis; DEA: data envelopment analysis; SFA: stochastic frontier analysis; LOS: length of stay.
